# LAPTM proteins in neurological disorders—Autophagy–lysosome dysfunction and therapeutic targets: A review

**DOI:** 10.17305/bb.2026.13624

**Published:** 2026-02-10

**Authors:** Bowen Wu, Zhenyan Niu, Yanfang Sui

**Affiliations:** 1 UWE College of Hainan Medical University, Haikou, China; 2 Department of Nuclear Medicine, Affiliated Haikou Hospital of Xiangya Medical College, Central South University, Haikou, China; 3 Department of Rehabilitation Medicine, Affiliated Haikou Hospital of Xiangya Medical College, Central South University, Haikou, China

**Keywords:** LAPTM proteins, autophagy-lysosome pathway, neurological disorders, therapeutic targets, gene therapy

## Abstract

Lysosomal-associated protein transmembrane (LAPTM) family members—LAPTM4A, LAPTM4B, and LAPTM5—regulate lysosomal integrity, autophagy–lysosome flux, lipid homeostasis, and immune signaling, pathways increasingly implicated in neurological disease. This review synthesizes structure–function evidence for LAPTM proteins and examines how their dysregulation contributes to Alzheimer’s and Parkinson’s disease, ischemia–reperfusion injury, and gliomas. Based on a targeted narrative analysis of primary and translational studies, we highlight that LAPTM proteins influence lysosomal acidification and membrane stability, endolysosomal trafficking, and ceramide/ion handling, thereby shaping protein aggregate clearance, oxidative stress responses, and microglia/macrophage polarization. Preclinical data link LAPTM5 to stroke outcomes via stress-kinase and lysosomal pathways, while LAPTM4A and LAPTM4B associate with glioma progression, immune evasion, and therapy resistance. Overall, LAPTM proteins represent promising biomarkers and therapeutic targets, warranting cell-type-resolved validation and central nervous system (CNS)-optimized delivery strategies, including gene therapy, small-molecule/degrader approaches, and multi-omics-guided patient stratification.

## Introduction

Neurological disorders, including neurodegenerative diseases, cerebrovascular events, brain tumors, and neuroinflammatory conditions, present a significant global health crisis characterized by complex etiologies and severe impairments in quality of life. The accelerating aging population contributes to the rising incidence of Alzheimer’s disease (AD) and Parkinson’s disease (PD), while aggressive gliomas result in poor prognoses and substantial patient burdens [1, 2]. Despite advances in molecular neuroscience, effective root-cause therapies remain elusive, highlighting the urgent need for innovative regulators of disease mechanisms to serve as biomarkers and therapeutic targets.

Lysosomes function as critical centers for cellular degradation and are essential for maintaining homeostasis in the nervous system. In post-mitotic neurons, the autophagy-lysosome pathways are crucial for clearing protein aggregates and damaged organelles, thereby sustaining neuronal survival and synaptic integrity [3]. Dysfunctions in lysosomal activity, which disrupt proteostasis, mitochondrial function, and energy metabolism, are characteristic features of neurodegeneration, supported by extensive preclinical evidence.

The lysosomal-associated protein transmembrane (LAPTM) family, which includes multi-pass membrane proteins found in lysosomes and late endosomes, has emerged as a promising yet underexplored area of research. Notably, LAPTM4B and LAPTM5 have been the focus of numerous studies [4, 5]. LAPTM4B contains lysosomal targeting signals that stabilize membranes, enhance autophagosome maturation, and improve stress adaptation [6, 7]. Its amplification in cancer promotes autophagy-mediated survival under metabolic and chemotherapy-induced stress and enhances epidermal growth factor receptor–phosphoinositide 3-kinase/protein kinase B (EGFR–PI3K/AKT) signaling pathways to foster cellular proliferation [6, 8]. Additionally, LAPTM4A regulates membrane transporters and modulates intracellular trafficking [9]. LAPTM5, which is abundant in immune cells, regulates inflammation through the activation of nuclear factor kappa-light-chain-enhancer of activated B cells (NF-κB) and mitogen-activated protein kinase (MAPK) pathways and cytokine release [10]. It also modulates the WW domain-containing E3 ubiquitin protein ligase 2 (WWP2)–phosphatase and tensin homolog (PTEN)–AKT pathway to influence B-cell apoptosis and peripheral tolerance [5, 11].

Emerging evidence suggests that members of the LAPTM family (LAPTM4A, LAPTM4B, LAPTM5) are implicated in neurological disorders through complementary mechanisms. Dysregulated LAPTM proteins contribute to impaired autophagy-lysosome flux—a prominent feature observed in preclinical models of AD and PD—leading to compromised clearance of pathogenic proteins and reduced neuronal viability [12]. In central nervous system (CNS) tumors, LAPTM4A promotes the immunosuppressive M2 polarization of tumor-associated macrophages (TAMs), facilitating glioma progression and invasion [13]. Similarly, LAPTM4B is associated with enhanced cell proliferation, chemoresistance, and poor clinical outcomes, positioning these proteins as critical therapeutic targets [6]. Furthermore, LAPTM5, which modulates microglial immunity, plays a crucial role in regulating neuroinflammation associated with various neuropathologies [14]. Some LAPTM isoforms also influence the mechanistic target of rapamycin (mTOR)/PI3K/NF-κB pathways, which govern metabolism, cellular stress responses, and cell fate decisions [15].

Although the roles of LAPTM proteins in cancer and immunology are well-established, their functions within neural contexts—including neuron, glial, and immune cell specificity, as well as links to causal pathology—remain underexplored. This gap necessitates rigorous validation to uncover potential biomarkers and therapeutic strategies. This review aims to integrate LAPTM functions across neurodegenerative disorders, cerebrovascular diseases, CNS tumors, and neuroinflammation; elucidate underlying mechanisms; and highlight translational strategies that bridge laboratory research and clinical applications for precision neurology.

## Molecular structure and functions of LAPTM proteins

**Figure 1.**
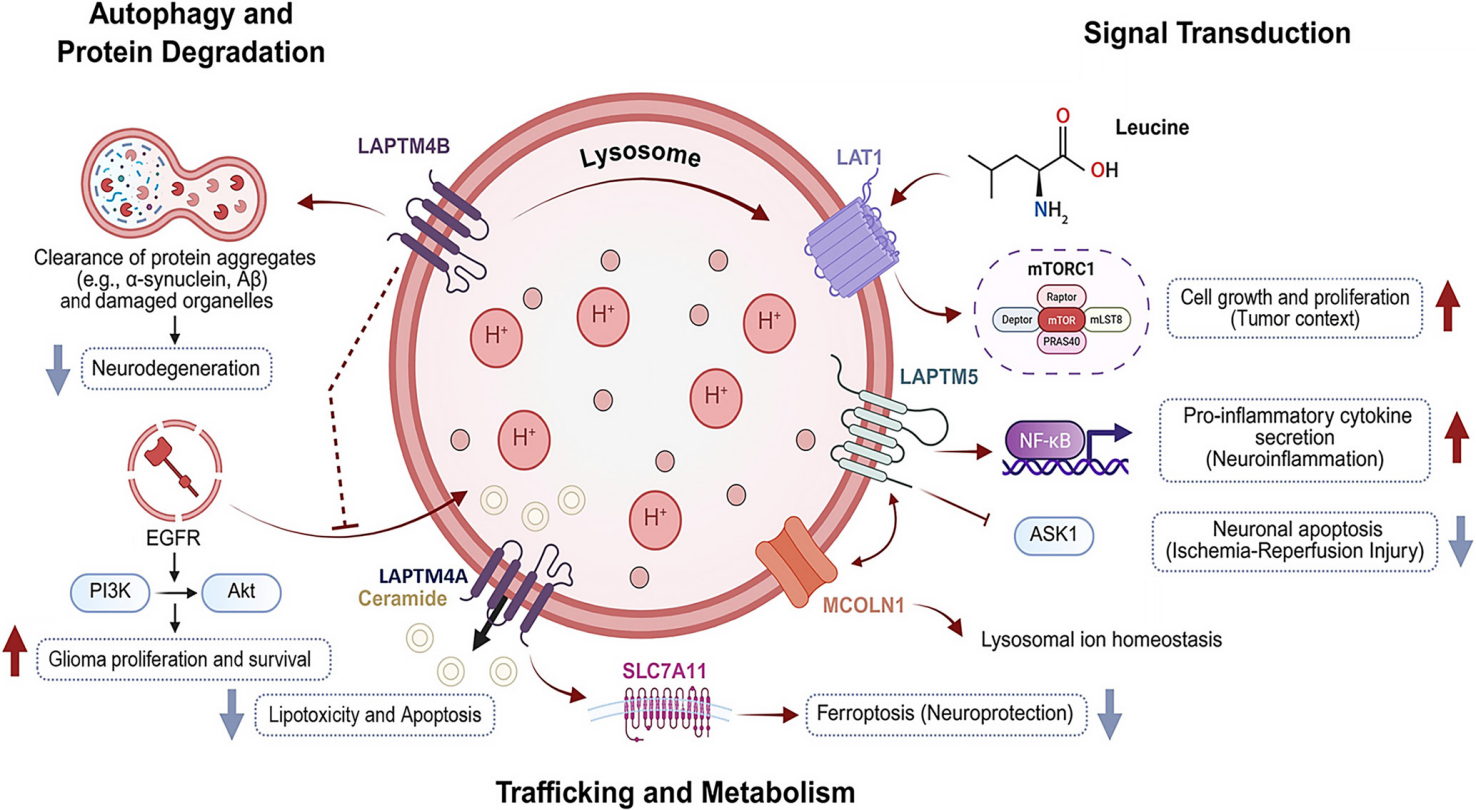
**Comprehensive roles of LAPTM4A, LAPTM4B, and LAPTM5 in lysosomal regulation and neurological disorders.** This schematic highlights how the three LAPTM family members prioritized in this review for their lysosomal/late endosomal localization and CNS relevance coordinate autophagy–lysosome flux, lysosomal acidification (V-ATPase–dependent H^+^ homeostasis), lipid handling (ceramide trafficking), and receptor/signaling networks. LAPTM4A/LAPTM4B support lysosomal integrity and proteostasis, influencing aggregate clearance (α-synuclein, Aβ) implicated in PD/AD, while LAPTM-linked trafficking sustains EGFR–PI3K–AKT signaling and LAT1–leucine–mTORC1 activation that promote growth in the tumor context. LAPTM5 couples lysosomal function to NF-κB–dependent cytokine programs and stress/apoptosis signaling (ASK1), shaping neuroinflammation and ischemia–reperfusion injury outcomes (Section 4), including glioma progression via TAM polarization. The symbols indicate the following: **↑** upregulation/activation, **↓** downregulation/inhibition, and **→** trafficking/activation. Abbreviations: LAPTM: Lysosomal-associated protein transmembrane; LAPTM4A: Lysosomal-associated protein transmembrane 4A; LAPTM4B: Lysosomal-associated protein transmembrane 4B; LAPTM5: Lysosomal-associated protein transmembrane 5; CNS: Central nervous system; V-ATPase: Vacuolar-type H+-ATPase; NF-κB: Nuclear factor kappa B; EGFR: Epidermal growth factor receptor; PI3K: Phosphoinositide 3-kinase; AKT: Protein kinase B; LAT1: L-type amino acid transporter 1; mTORC1: Mechanistic target of rapamycin complex 1; ASK1: Apoptosis signal-regulating kinase 1; TAM: Tumor-associated macrophage; PD: Parkinson’s disease; AD: Alzheimer’s disease; Aβ: Amyloid-beta.

**Figure 2.**
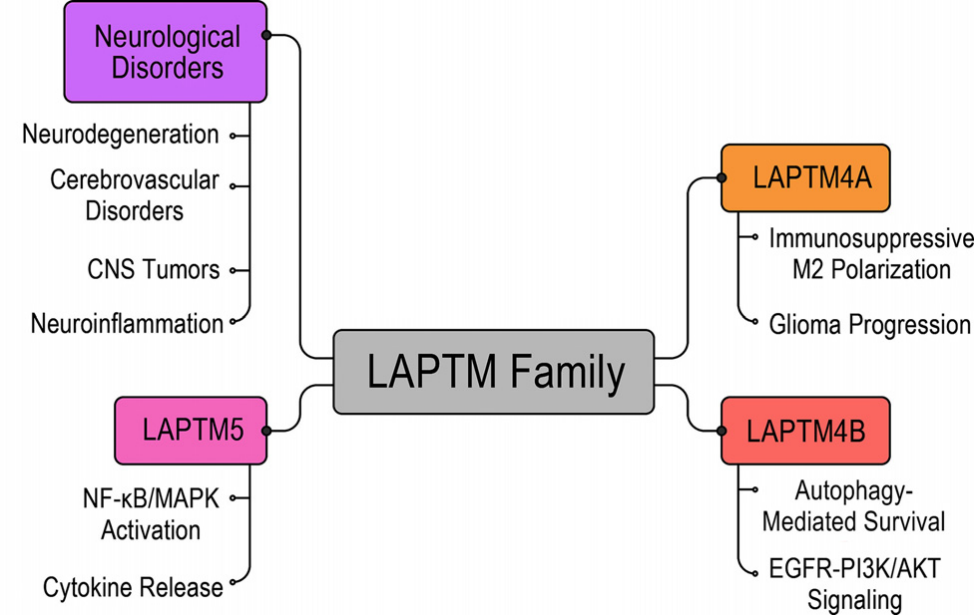
**LAPTM family proteins as convergent lysosomal regulators across neurological disorders.** This conceptual map places the LAPTM family as a central hub connecting lysosome-centered mechanisms to major CNS disease domains under the umbrella of “neurological disorders.” The central node (“LAPTM family”) integrates three prioritized, lysosome-associated members—LAPTM4A, LAPTM4B, and LAPTM5—shown as surrounding modules that represent protein-specific effector axes. LAPTM5 is positioned as a driver of neuroinflammatory programs via NF-κB/MAPK activation and downstream cytokine release; LAPTM4B is linked to neurodegeneration/proteostasis failure through sustained autophagy suppression and growth-factor signaling (EGFR–PI3K–AKT); and LAPTM4A is linked to CNS tumor biology by promoting immunosuppressive M2 tumor-associated macrophage polarization that supports glioma progression. Abbreviations: CNS: Central nervous system; LAPTM: Lysosomal-associated protein transmembrane; LAPTM4A: Lysosomal-associated protein transmembrane 4A; LAPTM4B: Lysosomal-associated protein transmembrane 4B; LAPTM5: Lysosomal-associated protein transmembrane 5; NF-κB: Nuclear factor kappa B; MAPK: Mitogen-activated protein kinase; EGFR: Epidermal growth factor receptor; PI3K: Phosphoinositide 3-kinase; AKT: Protein kinase B; TAM: Tumor-associated macrophage; M2: Alternatively activated (M2) macrophage polarization.

**Figure 3.**
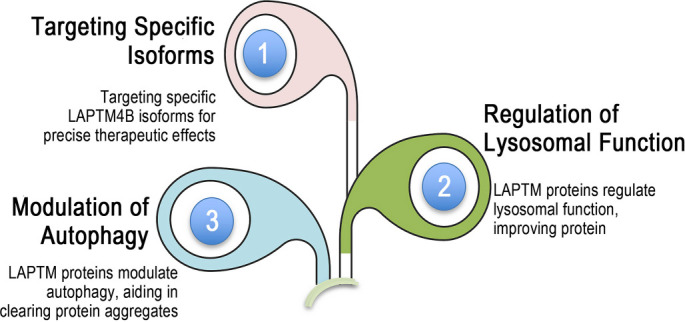
**Preclinical evidence for LAPTM-specific therapeutic strategies for neurological disorders.** This schematic summarizes three complementary therapeutic axes (1–3) proposed to counter LAPTM-driven lysosomal dysfunction across gliomas, neurodegeneration, and ischemia–reperfusion injury. (1) Isoform- and context-specific targeting: inhibition of LAPTM4B (e.g., siRNA approaches and lysosomotropic agents such as chloroquine) is conceptualized to restore lysosomal acidification/V-ATPase-linked function and normalize nutrient signaling (mTORC1) in PD and GBM, whereas LAPTM5 augmentation (e.g., AAV-mediated overexpression) is positioned to enhance autophagy–lysosome flux and neuroprotection in IRI. (2) Autophagy reactivation: suppression of LAPTM4A/LAPTM4B in disease-relevant models is depicted as a strategy to re-establish autophagosome–lysosome fusion and improve clearance of aggregation-prone proteins (α-synuclein, Aβ). (3) Restoration of lysosomal homeostasis: the third axis emphasizes correction of lysosomal pH and trafficking defects through LAPTM-regulated ubiquitin-dependent sorting and receptor trafficking (via UIM/PY motif–mediated interactions), thereby improving proteostasis and stress resistance across neurological contexts. Abbreviations: LAPTM: Lysosomal-associated protein transmembrane; LAPTM4A: Lysosomal-associated protein transmembrane 4A; LAPTM4B: Lysosomal-associated protein transmembrane 4B; LAPTM5: Lysosomal-associated protein transmembrane 5; siRNA: Small interfering RNA; AAV: Adeno-associated virus; V-ATPase: Vacuolar-type H+-ATPase; mTORC1: Mechanistic target of rapamycin complex 1; PD: Parkinson’s disease; GBM: Glioblastoma; IRI: Ischemia–reperfusion injury; Aβ: Amyloid-beta; UIM: Ubiquitin-interacting motif; PY: Proline-rich PY motif.

The LAPTM family consists of five evolutionarily conserved members: LAPTM4A, LAPTM4B, LAPTM4C, LAPTM5, and LAPTM5B. This review focuses on LAPTM4A, LAPTM4B, and LAPTM5 due to their predominant localization in lysosomes and late endosomes, well-characterized roles in regulating autophagy-lysosome pathways, and established relevance to neurological disorders, including gliomas, ischemia, and neurodegeneration, as supported by extensive primary literature. In contrast, LAPTM4C is primarily localized to the plasma membrane with limited lysosomal function, while LAPTM5B exhibits hematopoietic-restricted expression and lacks documented roles in neural contexts, justifying their exclusion from this synthesis. LAPTM4A, LAPTM4B, and LAPTM5 localize to lysosomal and late endosomal membranes, sharing dileucine motifs, PY domains, and ubiquitin-interaction motifs (UIMs) that facilitate interactions with ubiquitin ligases and endosomal sorting (Figures 1–3, Tables 1 and 2), thereby presenting therapeutic potentials. These three LAPTM proteins (LAPTM4A, LAPTM4B, LAPTM5) increasingly emerge as regulators of signal transduction, lipid metabolism, receptor trafficking, and autophagy—processes vital for maintaining neural homeostasis in the context of neurodegenerative lysosomal defects (see Figure 1) [16]. This section delineates their structural characteristics, trafficking pathways, and signaling roles, emphasizing interactions among neurons, glia, and microglia. The integration of LAPTM proteins with neurotrophic cues, amino acid sensing, and immune balance underscores their pivotal roles in brain physiology and pathology.

**Table 1:** Comparative molecular and functional profiles of neurologically relevant LAPTM family members

**Characteristic**	**LAPTM4A**	**LAPTM4B**	**LAPTM5**	**Evidence tier (1=Preclinical, 2=human correlation, 3=validated)**
Structure	Four-transmembrane protein	Four-transmembrane proto-oncogene	Five-transmembrane protein	1
Localization	Late endosomes/lysosomes	Late endosome/lysosome	Lysosomes	1
Function	Regulates endosomal sorting, lysosomal integrity, and lipid homeostasis	Exports lysosomal ceramides, acidifies lysosomes, matures autophagosomes	Modulates lysosomal ion homeostasis, amplifies pro-inflammatory responses	1
Role in Disease	Drives M2 polarization in gliomas, confers toxin resistance	Overexpressed in cancers, promotes tumor growth, chemoresistance	Fosters aggressive phenotypes in cancer, accelerates tubular senescence	1; 2
Neurological Relevance	High expression in gliomas, potential target for neurological disorders	Overexpression in glioblastoma, potential target for neurodegeneration	Proinflammatory and autophagic roles in microglia, potential therapeutic relevance	2; 1

Abbreviations: LAPTM: Lysosomal-associated protein transmembrane; LAPTM4A: Lysosomal-associated protein transmembrane 4A; LAPTM4B: Lysosomal-associated protein transmembrane 4B; LAPTM5: Lysosomal-associated protein transmembrane 5; M2: Alternatively activated (M2) macrophage polarization.

**Table 2:** Comparative overview of the LAPTM family with neurological inclusion criteria

**LAPTM protein**	**Chromosomal location**	**Lysosomal localization**	**Key domains**	**Primary neurological role**	**Exclusion rationale**
*LAPTM4A*	8q22.1	Yes (late endosomes)	PY, UIM, dileucine	Glioma TAM polarization, neuroinflammation	-
*LAPTM4B*	8q22.1	Yes (lysosomal membrane)	UIM, 4TMs	Neurodegeneration (PD/AD proteostasis), GBM progression	-
*LAPTM4C*	8q22.1	Plasma membrane	Limited lysosomal motifs	None documented	Non-lysosomal localization
*LAPTM5*	2p24.1	Yes (early endosomes)	FYVE, dileucine	Ischemia-reperfusion neuroprotection	-
*LAPTM5B*	2p24.1	ER/Golgi	Hematopoietic-specific	None documented	Non-neural expression

Abbreviations: LAPTM: Lysosomal-associated protein transmembrane; LAPTM4A: Lysosomal-associated protein transmembrane 4A; LAPTM4B: Lysosomal-associated protein transmembrane 4B; LAPTM4C: Lysosomal-associated protein transmembrane 4C; LAPTM5: Lysosomal-associated protein transmembrane 5; LAPTM5B: Lysosomal-associated protein transmembrane 5B; TAM: Tumor-associated macrophage; PD: Parkinson’s disease; AD: Alzheimer’s disease; GBM: Glioblastoma; PY: Proline-rich PY motif; UIM: Ubiquitin-interacting motif; FYVE: Fab1–YOTB–Vac1–EEA1 domain; 4TMs: Four transmembrane domains; ER: Endoplasmic reticulum.

### LAPTM4A

LAPTM4A, a four-transmembrane protein encoded by the LAPTM4A gene, localizes to late endosomes and lysosomes [17]. Its structural features include cytosolic N- and C-termini, hydrophobic transmembrane helices, dileucine/tyrosine motifs, and C-terminal PY domains that facilitate the recruitment of NEDD4-1 for ubiquitination and degradation. These characteristics are evolutionarily conserved and essential for membrane dynamics [17, 18].

LAPTM4A plays a crucial role in regulating endosomal sorting and maintaining lysosomal integrity by partnering with transporters such as human organic cation transporter 2 (hOCT2) to modulate cationic uptake, recycling, and detoxification [9]. In the context of lipid homeostasis, it post-transcriptionally fine-tunes globotriaosylceramide (Gb3) synthase, preventing lysosomal Gb3 accumulation and associated pathology [19]. In gliomas, LAPTM4A promotes M2 polarization of TAMs, contributing to immunosuppression, proliferation, and invasion [13]. Additionally, it confers resistance to toxins (e.g., Shiga toxin, ricin) through endosomal processing and ceramide-mediated membrane stabilization [20]. High expression levels of LAPTM4A are associated with advanced glioma grades, increased aggressiveness, and poor survival outcomes [21], often mediated through miRNA/circRNA axes. Regulatory RNAs—including microRNAs, long non-coding RNAs (lncRNAs), and circular RNAs (circRNAs)—fine-tune LAPTM expression post-transcriptionally, frequently through 3′UTR binding that suppresses LAPTM4A translation in glioma cells and exerts tumor-suppressive effects [21, 22]. However, these regulatory links remain unproven in neurological disorders, including tumors. There is no direct evidence linking miRNA/circRNA modulation of LAPTM4A/B/5 to autophagy defects in AD or PD, glioma tumor-associated macrophage polarization, ischemia, or neuroinflammation mechanisms. This gap underscores a critical frontier in research, suggesting that neural-specific RNA-LAPTM networks could illuminate biomarkers and therapeutic miRNA mimics/antagomirs for precision neurology [23]. Thus, the architecture of LAPTM4A underpins diverse roles in trafficking, metabolism, immunity, and oncology-related endolysosomal control.

### LAPTM4B

LAPTM4B, a four-transmembrane proto-oncogene, is overexpressed in various cancers, including hepatocellular carcinoma, where it serves as a prognostic marker [24]. Its cytosolic N- and C-termini, PY motifs for NEDD4 ubiquitin ligase binding, and two isoforms (notably the tumor-dominant LAPTM4B-35) facilitate localization to late endosomes and lysosomes, as well as lipid interactions (e.g., ceramides, PI(4,5)P2) [25–27].

LAPTM4B exports lysosomal ceramides independently of sphingomyelin synthase, preventing accumulation and subsequent cell death, while preserving lysosomal integrity [28]. It acidifies lysosomes, matures autophagosomes for fusion, and sustains flux to support tumor growth [29]. Furthermore, it recruits the L-type amino acid transporter 1–4F2 heavy chain complex (LAT1–4F2hc) to promote leucine/mechanistic target of rapamycin complex 1 (mTORC1)-driven proliferation under stress conditions [30].

Oncogenically, LAPTM4B enhances migration through integrin-β1/focal adhesion kinase (FAK)-AKT signaling [31], inhibits EGFR degradation to sustain signaling [27], stabilizes ribosomal protein S9 (RPS9)/signal transducer and activator of transcription 3 (STAT3) in leukemia [32], and alters extracellular vesicle sphingolipids for microenvironmental crosstalk [33]. LAPTM4B polymorphisms have been linked to increased susceptibility to gastric, colon, and ovarian cancers [34].

Transcription factors such as histone deacetylase 2 (HDAC2), ETS variant transcription factor 1 (ETV1), and transcription factor AP-4 (AP4) upregulate LAPTM4B expression in hepatocellular carcinoma, enhancing autophagy and cancer stemness [35, 36]. By stabilizing solute carrier family 7 member 11 (SLC7A11), LAPTM4B inhibits ferroptosis and mitigates lipid peroxidation [37]. In glioblastoma, elevated LAPTM4B-35 expression correlates with poor prognosis and promotes angiogenesis, with co-amplification alongside tyrosine 3-monooxygenase/tryptophan 5-monooxygenase activation protein zeta (YWHAZ) contributing to chemotherapy resistance [38, 39]. Moreover, LAPTM4B facilitates migration and invasion in various cancers, including hepatocellular carcinoma, lung adenocarcinoma, acute lymphoblastic leukemia, and breast cancer [36, 40–43].

In neurological contexts, LAPTM4B-35 overexpression is associated with glioblastoma angiogenesis, progression, and chemoresistance—paralleling neurodegenerative processes linked to ceramide dysregulation and lipofuscin accumulation (e.g., in neuronal ceroid lipofuscinosis and Lewy body dementia). This positions LAPTM4B as a potential target for neural tumors and lysosomal dysfunction, although neuron-specific validation is lacking. Overall, LAPTM4B’s structural attributes facilitate lysosomal metabolism, survival signaling, and oncogenic processes.

### LAPTM5

LAPTM5, or lysosomal-associated protein transmembrane 5, features a distinctive five-transmembrane topology with three PY motifs and a UIM, complemented by cytosolic loops and termini that promote supramolecular complexes and RING domain-mediated signaling [5]. These PY and UIM elements are critical for recruiting E3 ligases, such as Nedd4, to drive substrate ubiquitination and degradation [5, 44].

Functionally, LAPTM5 modulates lysosomal ion homeostasis through interactions with mucolipin-1 (MCOLN1), alleviating mucolipidosis type IV-like phenotypes [45]. In the context of innate immunity, LAPTM5 amplifies pro-inflammatory responses in macrophages by activating NF-κB and MAPK pathways, thereby enhancing cytokine production [10]. During T-cell activation, LAPTM5 promotes the lysosomal degradation of CD3 zeta chain (CD3^ζ^), suppressing T-cell receptor surface expression [46].

In oncology, LAPTM5 fosters aggressive phenotypes; for instance, FOXP3 upregulates LAPTM5 in breast cancer to enhance proliferation, migration, and invasion [47]. Elevated LAPTM5 expression has been implicated in the progression of clear cell renal cell carcinoma [48]. Clustered regularly interspaced short palindromic repeats (CRISPR) screens indicate that LAPTM5 confers lenvatinib resistance in hepatocellular carcinoma [49], while ZKSCAN5-SETD7-mediated activation of histone H3 lysine 4 trimethylation (H3K4me3) supports pancreatic ductal adenocarcinoma growth [50]. Similarly, LAPTM5 enhances venetoclax resistance in multiple myeloma through MCL-1 stabilization [51] and contributes to cisplatin resistance in non-small cell lung cancer by suppressing ferroptosis through cathepsin D [52].

Beyond cancer, LAPTM5 accelerates tubular senescence and fibrosis in kidney injury via WWP2/Notch1 signaling [53], activates autophagy in immune and inflammatory contexts [5], and marks M2 macrophage signatures in chronic rhinosinusitis [54]. In neurological contexts—particularly concerning microglia and neuroinflammation—LAPTM5’s pro-inflammatory and autophagic roles suggest therapeutic relevance, although direct validation in neurodegeneration, gliomas, or ischemia is still pending. Overall, LAPTM5’s architecture facilitates versatile control of lysosomal degradation, immune signaling, and pathology across a range of diseases.

## Roles of LAPTM proteins in nervous system homeostasis

Lysosomal dysfunction is a central pathological feature across various neurological disorders, including neurodegeneration, ischemic injury, brain tumors, and neuroinflammation [55]. The LAPTM family (LAPTM4A, LAPTM4B, LAPTM5) serves as pivotal regulators of lysosomal acidification, membrane trafficking, autophagy, and cellular degradation, positioning them as essential guardians of nervous system homeostasis. In neural tissues—where post-mitotic neurons and metabolically active glia experience relentless demands—LAPTM-mediated lysosomal integrity prevents toxic accumulation of proteins, lipids, and organelles that can precipitate pathology [56]. LAPTM proteins orchestrate endolysosomal trafficking and ion homeostasis through interactions with ubiquitin ligases and ion channels, supporting neuronal survival and glial functions [57]. In neurons, they facilitate autophagy-lysosome clearance of misfolded proteins to alleviate proteotoxic stress [58]. In glial cells, they balance inflammation and metabolic support to maintain tissue integrity [59]. Mouse models and human data link LAPTM dysregulation to ischemia-reperfusion injury, intracerebral hemorrhage, gliomas, and age-related hearing loss, with disruptions triggering apoptosis, inflammation, and circuit failure. This section examines the contributions of LAPTM proteins to neuronal and glial homeostasis, synthesizing emerging evidence regarding their mechanisms and implications for neurological health (Table 3).

**Table 3:** Functions of LAPTM protein in neuronal and glial homeostasis within the CNS

**Characteristic**	**Neurons**	**Glial cells**	**Evidence tier (1=preclinical, 2=human correlation, 3=validated)**
Function	Autophagy, ion homeostasis, protein clearance	Phagocytosis, cytokine modulation, myelin maintenance	1
LAPTM4A	Regulates cation uptake, mitigates mitophagy	Balances inflammation, correlates with poor glioma prognosis	1
LAPTM4B	Exports ceramides, sustains autophagic flux	Regulates exosome release, influences glioblastoma progression	1
LAPTM5	Drives autophagy, influences neuroimmune interactions	Amplifies inflammation, drives glial invasion	1

Abbreviations: CNS: Central nervous system; LAPTM: Lysosomal-associated protein transmembrane; LAPTM4A: Lysosomal-associated protein transmembrane 4A; LAPTM4B: Lysosomal-associated protein transmembrane 4B; LAPTM5: Lysosomal-associated protein transmembrane 5.

## Neurons

Post-mitotic neurons exhibit specialized architecture and possess high metabolic demands, relying on robust lysosomal systems for autophagy-mediated clearance of damaged organelles and proteins to maintain cellular homeostasis [60]. This review highlights LAPTM proteins (LAPTM4A, LAPTM4B, LAPTM5) as critical enhancers of lysosomal membrane stability and trafficking, thereby safeguarding neuronal integrity under neurological stressors. Notably, LAPTM5 deficiency in mouse models exacerbates cerebral ischemia/reperfusion injuries through increased c-Jun N-terminal kinase (JNK)/p38 mitogen-activated protein kinase (p38) signaling, impaired lysosomal function, and heightened apoptosis and inflammation, while its overexpression offers protective effects [60, 61]. In the context of intracerebral hemorrhage, LAPTM5 upregulation in peri-hematomal neurons modulates degradation pathways, suppresses apoptosis signal-regulating kinase 1 (ASK1)-mediated apoptosis, and prevents secondary injury [61]. Furthermore, LAPTM4A collaborates with hOCT2 transporters to regulate brain cation uptake, detoxification, and ion balance [9]. In PD models, miR-570-1 modulation of LAPTM4A governs mitochondrial-lysosomal crosstalk, mitigating excessive mitophagy and neuronal death [62]. Moreover, LAPTM4A may protect against prion fragment-induced apoptosis through STI571-stabilized lysosomes and glycosphingolipid homeostasis, maintaining membrane function [19, 63].

Similarly, LAPTM4B enhances neuronal resilience by exporting lysosomal ceramides to prevent apoptosis and sustain autophagic flux [28]. Mutations in LAPTM4B associated with neuronal ceroid lipofuscinosis disrupt proteostasis, leading to lipofuscin accumulation [64]. Additionally, LAPTM4B copy number variations in dementia with Lewy bodies impair α-synuclein degradation, thereby amplifying toxicity [65]. LAPTM4B also influences auditory neuron gene expression in age-related hearing loss [66].

LAPTM proteins contribute to neuronal ion homeostasis via interactions with mucolipin-1 (preventing storage disorders) and ubiquitin sorting (PY motifs), essential for circuit homeostasis. In peripheral immune cells, LAPTM5 promotes the lysosomal degradation of the T-cell receptor CD3^ζ^, suppressing activation and potentially modulating neuroimmune interactions during CNS inflammation [10, 67]. In spinocerebellar ataxia models, related transmembrane proteins are vital for maintaining lysosomal integrity and Purkinje neuron survival, underscoring the broader family’s critical role in neuronal maintenance [68].

In neuroblastoma, LAPTM5 induces spontaneous regression of neuronal progenitors through targeted lysosomal destabilization and autophagic cell death, illustrating its context-dependent role in neural lineage control. This mechanism is particularly relevant to gliomas, which are central to this review, where LAPTM proteins likely mediate neuronal-glial crosstalk, as indicated by prognostic correlations in low-grade gliomas and compensatory upregulation patterns during tumor progression [7, 10, 13]. These insights emphasize how dysregulation of LAPTM proteins amplifies neuronal vulnerability across ischemia-reperfusion injury, hemorrhagic damage, protein aggregation in degeneration, and oncogenic transformation. By optimizing lysosomal degradation, reducing stress-induced apoptosis, and preserving metabolic equilibrium, LAPTM proteins emerge as significant contributors to neuronal homeostasis, providing a compelling therapeutic rationale for the neurological disorders discussed in this review.

## Glial cells

Glial cells, including astrocytes, microglia, and oligodendrocytes, provide essential structural, metabolic, and immune support to neurons, with lysosomal functions underpinning phagocytosis, cytokine modulation, and myelin maintenance—crucial for nervous system homeostasis [69]. This modulation extends to microglia, where LAPTM4A balances pro-inflammatory and anti-inflammatory states disrupted by chemokine-like factor (CKLF)-induced mitophagy defects and lysosomal dysfunction, contributing to neuroinflammation [70]. Elevated LAPTM4A expression in gliomas correlates with poor prognosis, indicating compromised glial homeostasis during malignancy.

LAPTM4B regulates glial intercellular communication by controlling ceramide-induced exosome release within tumor microenvironments, influencing homeostasis and supporting glioblastoma progression through glial proliferation and angiogenesis. The LAPTM4B-35 isoform serves as a prognostic marker, with copy number gains conferring chemotherapy resistance similar to breast cancer—potentially extending to glial tumors [38, 71]. Related transmembrane protein 106B (TMEM106B) has been implicated in hypomyelinating disorders, suggesting a significant role for LAPTM4B in the maintenance of oligodendroglial myelin [72].

LAPTM5 is predominantly expressed in microglia, enhancing pro-inflammatory NF-κB/MAPK signaling to regulate immune homeostasis [10, 73]. LAPTM5 deficiency exacerbates glial inflammation during cerebral ischemia, compromising supportive functions [60]. Additionally, LAPTM5-CD40 interactions in glioblastoma drive glial invasion and temozolomide resistance [74]. Other functions include CD1e-mediated antigen presentation in glial endosomes and contributions to cochlear glial networks in age-related hearing loss [75].

In astrocytes, LAPTM proteins likely facilitate lactate shuttling, cholesterol balance, and aquaporin-4 interactions for water-ion homeostasis—vital neuronal support disrupted in gliomas [76]. During neurodegeneration, LAPTM5 enhances glial phagocytosis of debris, preserving tissue integrity. These mechanisms align with LAPTM5’s M2 signatures in resolving inflammation [5] and suggest broader glial activation patterns in brain tumors.

The diverse roles of LAPTM proteins in glial cells are directly relevant to neurological disorders. LAPTM5’s proliferative effects in renal cell carcinoma models suggest analogous activation patterns in glioma-associated glia, promoting tumor-supportive phenotypes [48]. LAPTM4B’s regulation of inositol status implies contributions to neural signaling homeostasis critical for circuit integrity [27]. In frontotemporal lobar degeneration, related TMEM proteins highlight lysosomal dysfunction’s role in glial pathology, while interactions with the semaphorin family underscore glial-neuronal crosstalk. Neurotrophins supporting energy homeostasis likely involve LAPTM-modulated glial metabolism, alongside lysophospholipid receptors governing glial motility and differentiation. Innate lymphoid cells interfacing with the nervous system further implicate LAPTM in glial immune homeostasis, while N-methyl-D-aspartate (NMDA)-independent long-term potentiation in glial-neuronal circuits may depend on LAPTM-mediated lysosomal stability. Lysosome-associated membrane protein (LAMP) proteins’ roles in neurite outgrowth suggest analogous functions for LAPTM in glial process extension, and mucolipidosis type IV demonstrates disruptions in glial ion channels due to LAPTM-mucolipin interactions [77].

In summary, LAPTM proteins ensure glial lysosomal efficiency, regulate inflammation, and facilitate metabolic crosstalk, with dysregulation heightening vulnerability across gliomas, ischemia, neurodegeneration, and neuroinflammation. Their therapeutic modulation offers promise for restoring glial-neuronal balance in neurological diseases.

### Dysregulation of LAPTM proteins in neurological disorders

As discussed in previous sections regarding molecular functions and cellular homeostasis, LAPTM proteins (LAPTM4A, LAPTM4B, LAPTM5) serve as crucial mediators when dysregulated in neurological pathologies. Their interactions with ubiquitin ligases, ion channels, and essential signaling pathways such as NF-κB and MAPK significantly impact neuroinflammation, apoptosis, and tumor progression [14]. For example, LAPTM4A and LAPTM4B are upregulated in gliomas, contributing to tumor aggressiveness, therapeutic resistance, and the formation of immunosuppressive microenvironments [21].

The structural characteristics of these proteins—multiple transmembrane domains and PY motifs—facilitate lysosomal localization and interactions with ceramides and transporters, thereby exacerbating disease progression [73]. In neurodegenerative contexts, LAPTM5 is implicated as a potential risk gene, co-expressed with established AD loci within microglial networks, while LAPTM4A regulates mitochondrial-lysosomal crosstalk in Parkinson’s models, influencing neuronal death [62]. Clinical evidence further highlights the pathological significance of LAPTM proteins, as their expression levels correlate robustly with prognosis in low-grade gliomas (diagnostic utility) and various CNS disorders [78]. Consequently, LAPTM dysregulation encompasses ischemic, oncogenic, degenerative, and inflammatory processes—unifying lysosomal dysfunction as a common hallmark amenable to targeted interventions. This section systematically examines LAPTM dysregulation across ischemia-reperfusion injury, nervous system tumors, neurodegenerative diseases, and neuroinflammatory conditions, elucidating molecular mechanisms and clinical implications (Table 4).

**Table 4:** Contributions of LAPTM protein in major neurological disorders

**Characteristic**	**Ischemia-reperfusion injury**	**Nervous system tumors**	**Neurodegenerative diseases**	**Neuroinflammation**
LAPTM4A	Bolsters resilience, promotes M2 polarization	Loss disrupts M2 polarization, biomarker	Protective, governs neuronal death	Reduces NF-κB, attenuates activation
LAPTM4B	Exports ceramides, sustains autophagy	Drives proliferation, angiogenesis	No specific role reported to date	Contributes via ceramide signaling
LAPTM5	Deficiency worsens outcomes, overexpression protects	Influences microglial clearance failure	Regulator correlated with disease severity	Drives NF-κB, escalates cytokine release

Abbreviations: LAPTM: Lysosomal-associated protein transmembrane; LAPTM4A: Lysosomal-associated protein transmembrane 4A; LAPTM4B: Lysosomal-associated protein transmembrane 4B; LAPTM5: Lysosomal-associated protein transmembrane 5; M2: Alternatively activated (M2) macrophage polarization; NF-κB: Nuclear factor kappa B.

## Ischemia-reperfusion injury

Building upon the established roles of LAPTM proteins in neuronal and glial homeostasis discussed in earlier sections, ischemia-reperfusion injury (IRI)—most commonly associated with stroke—represents a critical domain where lysosomal integrity serves as a protective mechanism against oxidative stress, inflammation, and apoptosis. LAPTM5 deficiency significantly worsens cerebral ischemia/reperfusion (I/R) outcomes in mouse models, resulting in enlarged infarct sizes, exacerbated neurological deficits, and elevated pro-inflammatory cytokines through enhanced JNK/p38 signaling and compromised lysosomal function [60]. Conversely, LAPTM5 overexpression provides robust neuroprotection by inhibiting ASK1-mediated apoptosis, preserving lysosomal acidification, and maintaining autophagic flux to prevent cathepsin release and secondary tissue damage [61].

LAPTM4A enhances I/R resilience through dual actions on glial immunity and neuronal ion balance. In chemokine-like factor 1 (CKLF1)-induced models, LAPTM4A deficiency impairs mitophagy, thereby reducing microglial pro-inflammatory activation and associated neuroinflammation. Restoration of PTEN-induced putative kinase 1 (PINK1)/Parkin flux during neuroinflammation models mitigates CKLF1-induced mitochondrial accumulation in BV2 microglia, attenuating pro-inflammatory activation [70]. This aligns with its promotion of M2 microglial polarization, fostering a reparative environment during reperfusion, complemented by hOCT2 transporter interactions that alleviate ischemic calcium overload [9, 13]. The upregulation of LAPTM4A in I/R-affected regions correlates with improved survival, underscoring its therapeutic relevance.

LAPTM4B contributes to ceramide homeostasis by exporting lysosomal ceramides to prevent lipid-induced neuronal apoptosis and sustain autophagic clearance [28]. Under hypoxic stress simulating I/R in neuroblastoma models, LAPTM4B regulates exosome-mediated aggregate removal, while its deficiency leads to ceramide accumulation, mitochondrial dysfunction, and oxidative stress [71].

Collectively, LAPTM proteins orchestrate lysosomal protection against I/R pathology, with clinical stroke data linking reduced LAPTM5 levels to adverse prognosis and indicating potential for gene therapy or agonist interventions. LAPTM5-CD1e interactions stabilize membranes and enhance antigen presentation to resolve post-ischemic inflammation, while LAPTM4A’s regulation of valosin-containing protein (VCP)/V-type proton ATPase subunit d1 (ATP6V0D1) in stress models aligns with I/R demands—positioning this protein family as vital protectors against acute neurological threats.

## Nervous system tumors

Extending the patterns of dysregulation observed in ischemia-reperfusion injury, nervous system tumors—particularly gliomas and glioblastomas—illustrate the pathological impact of LAPTM proteins through disrupted autophagy, immune evasion, and unchecked proliferation that characterize their aggressive growth and therapeutic resistance. Notably, loss of LAPTM4A disrupts M2 polarization of TAMs in glioblastoma, shifting the microenvironment toward immune activation and significantly enhancing the efficacy of anti-PD-1 therapy while reducing TAM infiltration and tumor proliferation [13, 79]. As previously noted, LAPTM4A correlates with prognosis across glioma grades, with elevated expression strongly associated with histological aggressiveness, advanced tumor stages, and poor patient outcomes [80].

Similarly, LAPTM4B serves as a prognostic marker in glioblastoma, where the LAPTM4B-35 isoform drives proliferation, angiogenesis, and poor outcomes via elevated vascular endothelial growth factor signaling and neovascularization [38]. This isoform facilitates ceramide-mediated exosome release to orchestrate communication between the tumor and its microenvironment, while intriguingly, the combination of LAPTM4B disruption with KPT330 enhances sensitivity to olaparib through lysosomal compromise, revealing potential therapeutic vulnerabilities [38, 81]. Clinical data position LAPTM expression as a predictor of recurrence in gliomas, with LAPTM4A knockdown models exhibiting reduced invasion and heightened immunotherapy responses.

Mechanistically, LAPTM4A promotes M2 TAM polarization via NF-κB signaling, establishing immunosuppressive niches, while its regulation of miR-5701 in Parkinson’s stress responses highlights intriguing parallels between neurodegenerative and oncogenic processes. The validated prognostic role of LAPTM4B and LAPTM5’s influence on microglial clearance during apoptosis further solidify the family’s significance in CNS oncobiology. Collectively, these findings—linking molecular drivers to clinical utility—position LAPTM proteins as compelling therapeutic targets for gliomas, unifying pathological themes and paving the way for precision interventions explored in subsequent sections.

## Neuro degenerative diseases

Beyond the acute ischemic and oncogenic pathologies previously discussed, LAPTM proteins exert significant influence across chronic neurodegenerative diseases—specifically AD and PD—which are central to this review’s focus on neurological disorders. In AD, LAPTM5 emerges as a microglia-centric regulator closely associated with disease severity, functioning through gene networks intertwined with TYROBP and CSF1R to orchestrate neuroinflammatory cascades [82]. Genetic variability within LAPTM5 further modulates responses to amyloid-beta, directly affecting susceptibility to AD risk [83]. Longevity studies identify LAPTM5 as a potential risk gene, demonstrating co-expression with established immune regulators ITGAM and LILRB4 within microglial compartments [84]. Integrated multi-omics analyses illustrate how dietary factors, APOE genotypes, and sex-specific influences converge on LAPTM5-associated immune networks in AD models, with weighted co-expression network analysis confirming its central role in driving inflammatory pathogenesis [82]. LAPTM5’s neuroinflammatory signature extends to neuropathic pain, where it occupies a crucial position within rat dorsal horn gene expression profiles, engaging protein-protein interaction networks with CD68 and C1QC to perpetuate chronic constriction injury-induced pain through sustained neuroinflammatory signaling [85]. In PD, LAPTM4A assumes a protective yet dysregulatable role, with miR-5701-mediated modulation influencing neuronal death susceptibility via altered VCP and LAPTM4A transcript dynamics. This positions LAPTM4A at the mitochondrial-lysosomal interface, where mitophagy defects lead to α-synuclein accumulation and dopaminergic cell death characteristic of PD pathology [86]. These findings illuminate LAPTM proteins as molecular linchpins bridging lysosomal dysfunction, microglial activation, and proteotoxic stress across AD and PD—offering mechanistic continuity with gliomas and ischemia while underscoring untapped therapeutic potential in neurodegeneration.

### Neuroinflammation and related conditions

LAPTM4A-mediated microglial polarization has been observed in both preclinical models and human correlative studies. *In vitro* experiments utilizing BV2 microglia and primary rat cultures demonstrate that LAPTM4A siRNA knockdown significantly decreases NF-κB nuclear translocation and the secretion of pro-inflammatory cytokines such as IL-6 and tumor necrosis factor alpha (TNF-α). *In vivo*, heterozygous LAPTM4A knockout in an lipopolysaccharide (LPS)-induced neuroinflammation mouse model results in reduced microglial activation, as indicated by diminished ionized calcium-binding adapter molecule 1 (Iba1) staining, and mitigates cognitive deficits in the Morris water maze [13]. Analysis of brain tissue from AD shows specific upregulation of LAPTM4A in Iba1+ microglia, correlating with disease severity. Currently, there are no interventional data from human studies apart from genome-wide association study (GWAS), highlighting a significant translational gap in this pathway [14, 84].

Neuroinflammation serves as a unifying pathological mechanism across the neurological disorders discussed in this review, with LAPTM proteins—particularly LAPTM5—playing a crucial role in orchestrating microglial activation, cytokine cascades, and immune homeostasis. When dysregulated, these processes can exacerbate tissue damage in conditions such as ischemia, gliomas, and neurodegeneration [14]. LAPTM5 is particularly prominent in this context, driving NF-κB and MAPK signaling in microglia and macrophages, which escalates pro-inflammatory cytokine release and perpetuates chronic neuroinflammatory states that contribute to neuronal loss and glial dysfunction. This aligns with earlier findings: LAPTM5 deficiency exacerbates ischemic glial inflammation [60], while its interactions with CD40 in glioblastoma create immune microenvironments that are resistant to temozolomide treatment [74].

In neurodegenerative contexts, LAPTM5 co-expression with TYROBP/CSF1R in AD microglia sustains amyloid-responsive inflammation, reflecting its pivotal role in neuropathic pain networks alongside CD68/C1QC within dorsal horn profiles [87]. LAPTM4A complements this by facilitating M2 TAM polarization in gliomas—its loss shifts the immune response towards antitumor activity—while CKLF1-induced mitophagy defects demonstrate its regulatory influence on neuroinflammatory balance [13]. Additionally, LAPTM4B indirectly contributes through ceramide-mediated exosome signaling that shapes inflammatory tumor microenvironments [71].

These mechanisms have practical implications: LAPTM5-CD1e partnerships modulate antigen presentation to resolve post-ischemic inflammation, and parallels with chronic rhinosinusitis M2 signatures suggest potential for resolution. Dysregulation thus perpetuates a vicious cycle of microglial priming, ineffective debris clearance, and circuit collapse across the conditions discussed herein. Targeting LAPTM-driven neuroinflammation presents a convergent therapeutic strategy, bridging acute and chronic pathologies toward precision interventions.

## Therapeutic potential of targeting LAPTM proteins

Building on the pathological dysregulation of LAPTM proteins across ischemia, gliomas, neurodegeneration, and neuroinflammation discussed in Section 4, therapeutic strategies targeting this protein family hold transformative potential for the neurological disorders central to this review. LAPTM4A, LAPTM4B, and LAPTM5 regulate autophagy-lysosome flux, lipid homeostasis, ion balance, and immune responses—pathways amenable to precise modulation. Their cell-type-specific roles position LAPTM proteins as ideal candidates for gene therapy, small-molecule interventions, immunotherapy combinations, and biomarker-driven care, as elaborated in the following subsections [88] (Tables 5-6).

**Table 5:** The therapeutic potential of targeting LAPTM proteins in neurological disorders

**Characteristic**	**Gene therapy and small-molecule modulators**	**Immunotherapy synergies and drug combinations**	**LAPTM as diagnostic and prognostic biomarkers**
Targeting strategy	Modulating proteins directly or enhancing gene therapy delivery	Combining LAPTM inhibitors with other therapies	Using LAPTM expression to stratify risk
Evidence	LAPTM5 overexpression reduces infarct size	LAPTM4A knockout synergizes with anti-PD-1 therapy	LAPTM4A overexpression stratifies glioma grade
Potential applications	Enhancing degradation of toxic protein aggregates	Amplifying immune checkpoint inhibitors	Enabling real-time progression tracking
Challenges	Specific small-molecule modulators not yet described	Requires clinical translation	Requires further validation in CNS disorders

Abbreviations: LAPTM: Lysosomal-associated protein transmembrane; LAPTM4A: Lysosomal-associated protein transmembrane 4A; LAPTM5: Lysosomal-associated protein transmembrane 5; PD-1: Programmed cell death protein 1; CNS: Central nervous system.

**Table 6:** Dysregulation of LAPTM protein in neurological disorders

**Characteristic**	**LAPTM4A**	**LAPTM4B**	**LAPTM5**	**Evidence tier (1=preclinical, 2=human correlation, 3=validated)**
Role in gliomas	Drives M2 polarization, promotes immunosuppression, proliferation, and invasion	Drives proliferation, chemoresistance, and poor outcomes	CD40 interactions drive glial invasion and temozolomide resistance	1; 2
Role in neurodegeneration	Governs mitochondrial- lysosomal crosstalk, mitigating neuronal death	Disrupts proteostasis with lipofuscin buildup, impairs α-synuclein degradation	Putative risk gene, co-expressed with Alzheimer’s disease loci	1; 2
Role in ischemia-reperfusion injury	Bolsters resilience through glial immunity and neuronal ion balance	No direct role reported to-date	Deficiency worsens outcomes, overexpression confers neuroprotection	1
Role in neuroinflammation	Balances pro- and anti-inflammatory states in microglia	No direct role reported to date	Amplifies pro-inflammatory responses in macrophages	1; 2
Other roles	Regulates endosomal sorting and lysosomal integrity	Exports lysosomal ceramides, acidifies lysosomes, matures autophagosomes	Modulates lysosomal ion homeostasis, promotes lysosomal degradation of CD3^ζ^	1

Abbreviations: LAPTM: Lysosomal-associated protein transmembrane; LAPTM4A: Lysosomal-associated protein transmembrane 4A; LAPTM4B: Lysosomal-associated protein transmembrane 4B; LAPTM5: Lysosomal-associated protein transmembrane 5; M2: Alternatively activated (M2) macrophage polarization; α-synuclein: Alpha-synuclein; CD3^ζ^: CD3 zeta chain.

### Gene therapy and small-molecule modulators

While specific small-molecule modulators targeting the LAPTM family for gene therapy have yet to be identified, their integral role in lysosomal function presents a promising future direction [89]. This opens the theoretical possibility of developing novel treatments for various diseases, including neurological conditions, by either directly modulating these proteins or enhancing gene therapy delivery through their associated pathways. Evidence-based interventions tested in LAPTM models demonstrate clear therapeutic feasibility. Mechanistically, LAPTM proteins regulate lysosomal function and autophagy, which are critical for cellular clearance processes that fail in many neurological diseases. Targeting these proteins could enhance the degradation of toxic protein aggregates, representing a promising therapeutic avenue [90]. The foundational roles of LAPTM proteins in lysosomal acidification, membrane stability, and autophagosome-lysosome fusion underscore their potential in neurodegeneration and ischemia. Notably, LAPTM5 overexpression in cerebral ischemia-reperfusion mouse models significantly reduces infarct size, improves neurological deficit scores, and suppresses pro-inflammatory cytokines through ASK1 pathway inhibition, while LAPTM5 knockdown results in worse outcomes—establishing the feasibility of bidirectional modulation [60]. Similarly, LAPTM4A knockout in glioma models inhibits tumor-associated macrophage M2 polarization and reduces invasion, providing direct proof-of-principle for genetic intervention strategies [13].

Preclinical evidence positions LAPTM proteins (4A/B/5) as promising therapeutic candidates for neurological diseases, warranting further translational studies. Furthermore, neural adeno-associated virus (AAV) vectors, validated delivery platforms for CNS-specific applications, enable targeted LAPTM modulation—such as LAPTM5 inhibition in stroke-affected neurons to restore lysosomal flux, or LAPTM4A silencing in glioma-associated macrophages to reprogram immunosuppressive polarization. CRISPR/Cas9 screens confirm the roles of LAPTM4B and LAPTM5 in chemoresistance, while proteolysis-targeting chimera (PROTAC) degraders and high-throughput screening of transmembrane ligand pockets offer scalable strategies to restore lysosomal homeostasis across neurodegeneration, ischemia, and neuroinflammation [39, 91, 92]. These platforms facilitate the translation of validated preclinical mechanisms into feasible clinical applications for LAPTM-targeted neurological therapies.

### Immunotherapy synergies and drug combinations

Strategies targeting LAPTM4B inhibition present tiered evidence across various models. *In vitro* studies demonstrate that chloroquine and bafilomycin A1 effectively reverse the LAPTM4B-mediated displacement of V-ATPase in human embryonic kidney 293 cells (HEK293) and the human neuroblastoma cell line SH-SY5Y. This reversal restores lysosomal acidification and enhances autophagic flux [93, 94]. *In vivo* preclinical trials show that chloroquine administration in 1-methyl-4-phenyl-1,2,3,6-tetrahydropyridine (MPTP) mouse models of PD reduces dopaminergic neuronal loss in the substantia nigra pars compacta (SNc) and enhances motor function [95]. Observational studies in humans reveal a correlation between elevated LAPTM4B expression and poorer progression-free survival in glioblastoma (GBM) cohorts, although no interventional trials have been conducted [38, 96]. Direct LAPTM4B knockdown using siRNA in glioma cell lines [29] or AAV-shRNA in glioma xenograft models, similar to TIMM44 (a LAPTM-like glioma marker) [97], confirms its therapeutic potential but necessitates clinical translation.

Empirical evidence for LAPTM immunotherapy synergies supports their clinical applicability. LAPTM4B-35 serves as a novel prognostic factor for GBM [38]. In GL261 GBM mouse models, LAPTM4A knockout synergizes with anti-PD-1 therapy, resulting in significant tumor volume reduction compared to anti-PD-1 monotherapy, alongside improved survival through the repolarization of TAMs characterized by M2 marker expression [98]. LAPTM4A deletion also inhibits tumor-associated microglia polarization, dismantling immunosuppressive niches and enhancing immune effector infiltration [13]. These findings validate LAPTM4A as an immunomodulatory target in gliomas.

Validated immune signaling mechanisms support broader combinatorial strategies. LAPTM5 drives NF-κB/MAPK pro-inflammatory activation in microglia and macrophages, while LAPTM5-CD40 interactions in GBM confer resistance to temozolomide [74], establishing dual immune-metabolic vulnerabilities suitable for checkpoint inhibition. The dominance of M2 TAMs in LAPTM4A creates exploitable immunosuppressive microenvironments that can be reversed through targeted deletion.

High-potential combination therapies leverage these validated mechanisms. Combining LAPTM inhibitors with mTOR/NF-κB antagonists promises synergistic neuroprotection, as demonstrated in ischemia (where LAPTM5 overexpression reduces glial inflammation) and extending to chronic neuroinflammation. LAPTM modulation enhances the efficacy of immune checkpoint inhibitors, chimeric antigen receptor T-cell (CAR-T) cell therapy [99], and oncolytic viruses against refractory CNS tumors [100], while multi-target strategies position LAPTM as a linchpin for next-generation immunotherapies across the spectrum of gliomas, ischemia, and neuroinflammation.

### LAPTM as diagnostic and prognostic biomarkers

Prognostic correlations observed in clinical cohorts underscore the potential diagnostic utility of LAPTM proteins. Elevated LAPTM4A expression robustly stratifies glioma grade and prognosis, with protein levels correlating with histological aggressiveness and chemotherapy resistance across The Cancer Genome Atlas (TCGA)/lower-grade glioma (LGG)/GBM cohorts. Similarly, LAPTM4B-35 predicts GBM recurrence and angiogenesis [38], establishing validated mRNA and protein thresholds for risk assessment and treatment monitoring.

Preclinical biomarker correlations extend into neurodegeneration. LAPTM5 exhibits strong co-expression with AD microglial risk genes in postmortem brain tissue, positioning it as a candidate biomarker for neuroinflammatory burden across disease stages [101]. Serum LAPTM4B demonstrates significant sensitivity and specificity for breast cancer detection [102], suggesting potential utility in cerebrospinal fluid and blood tests for CNS malignancies and neurodegenerative disorders [103].

Emerging applications in precision medicine build upon these validated correlations. Non-invasive positron emission tomography (PET) probes visualizing LAPTM4B in hepatocellular carcinoma indicate potential extensions for CNS tumors [43], while blood and CSF LAPTM profiling enables real-time tracking of disease progression. Machine learning models integrating LAPTM polymorphisms, expression dynamics, and clinical variables promise refined risk stratification across gliomas, stroke outcomes, and dementia progression [104]. Longitudinal monitoring of LAPTM expression links trajectory changes to therapeutic response, facilitating true precision medicine across neurological disorders.

### Mechanistic parallels: cancer insights informing neurological research

**Figure 4.**
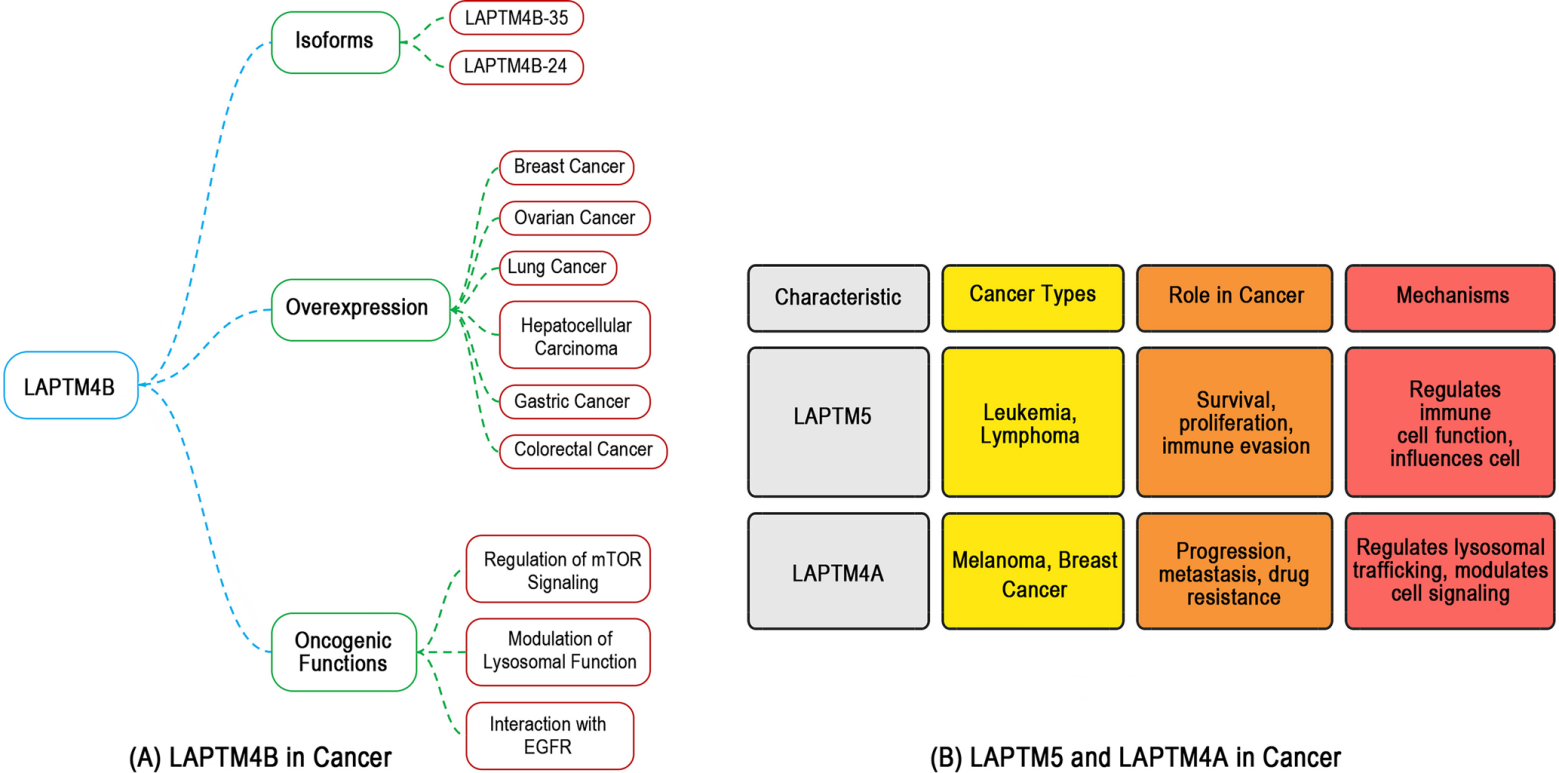
**LAPTM4A/B/5 mechanistic parallels from cancer research informing CNS therapeutic strategies.** Cancer literature is summarized as a translational framework for LAPTM-driven lysosomal reprogramming with relevance to neurological disease. (A) LAPTM4B: major isoforms (LAPTM4B-35, LAPTM4B-24) and broad tumor overexpression are linked to oncogenic functions via altered lysosomal homeostasis, including mTORC1-associated signaling, autophagy suppression, and V-ATPase–linked acidification changes, providing mechanistic parallels to proteostasis failure in PD/AD. (B) LAPTM5 and LAPTM4A: LAPTM5 is associated with hematologic malignancies (leukemia/lymphoma) through immune-regulatory signaling, whereas LAPTM4A is linked to melanoma/breast cancer through lysosomal trafficking programs that support progression and therapy resistance and conceptually align with TAM-related immunosuppression relevant to CNS tumors. Abbreviations: CNS: Central nervous system; LAPTM: Lysosomal-associated protein transmembrane; LAPTM4A: Lysosomal-associated protein transmembrane 4A; LAPTM4B: Lysosomal-associated protein transmembrane 4B; LAPTM5: Lysosomal-associated protein transmembrane 5; mTORC1: Mechanistic target of rapamycin complex 1; V-ATPase: Vacuolar-type H+-ATPase; PD: Parkinson’s disease; AD: Alzheimer’s disease; TAM: Tumor-associated macrophage.

**Figure 5.**
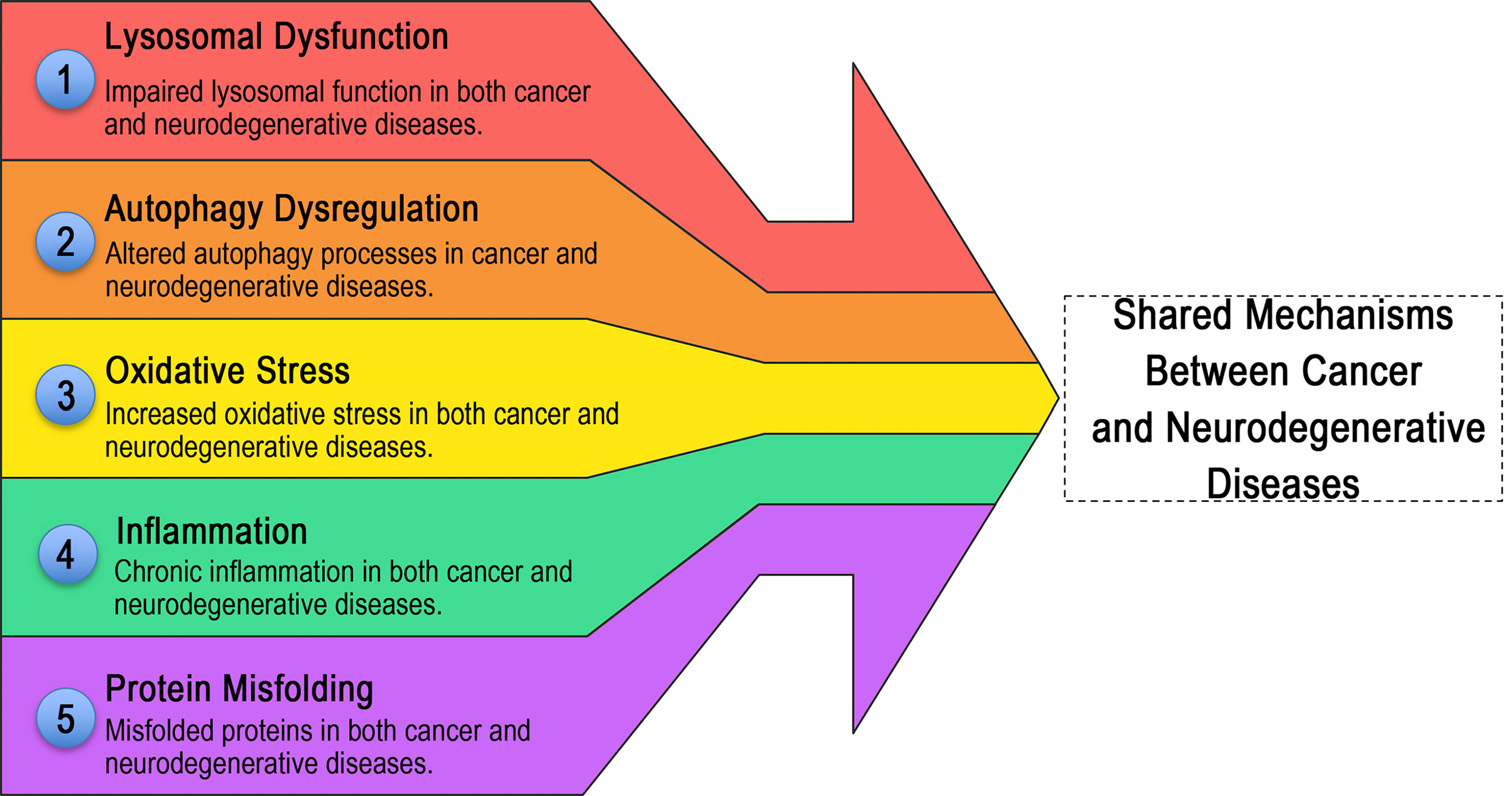
**Converging lysosomal pathways linking cancer and neurodegenerative disease mechanisms, with LAPTM proteins as therapeutic convergence points.** In line with the “mechanistic parallels” framework, this schematic summarizes five shared pathological hallmarks through which cancer-derived insights into LAPTM-driven lysosomal reprogramming can guide neurological translation. The arrow depicts convergence across (1) lysosomal dysfunction, including LAPTM4B-associated perturbation of V-ATPase–linked acidification reported in tumors and conceptually paralleling acidification failure implicated in PD/AD; (2) autophagy dysregulation, where LAPTM4A/LAPTM4B-dependent changes in autophagy–lysosome flux align with aggregate accumulation (α-synuclein, Aβ); (3) oxidative stress, highlighting that LAPTM-linked trafficking/lysosomal impairment can amplify redox vulnerability, relevant to ischemia–reperfusion injury; (4) inflammation, where LAPTM-mediated NF-κB programs in tumor-associated myeloid cells mirror microglia-driven neuroinflammatory states; and (5) protein misfolding, emphasizing ubiquitin-dependent sorting defects and proteostasis collapse as a common endpoint. Together, these hallmarks position LAPTM proteins as actionable nodes bridging cancer biology and neurological disease mechanisms. Abbreviations: LAPTM: Lysosomal-associated protein transmembrane; V-ATPase: Vacuolar-type H+-ATPase; PD: Parkinson’s disease; AD: Alzheimer’s disease; NF-κB: Nuclear factor kappa B; ALS: Amyotrophic lateral sclerosis; Aβ: Amyloid-beta.

Cancer research elucidates conserved lysosomal mechanisms that highlight LAPTM protein dysregulation in neurological disorders, serving as translational blueprints. LAPTM4A, LAPTM4B, and LAPTM5 exhibit parallel lysosomal reprogramming across tumors and neuropathologies, where insights from cancer research directly inform therapeutic strategies for neural disorders (Figures 4 and 5).

LAPTM4B-driven lysosomal dysfunction in hepatocellular carcinoma (HCC) and GBM—characterized by mTORC1 hyperactivation, macroautophagy suppression, and V-ATPase displacement—mirrors the overexpression of LAPTM4B observed in PD with α-synuclein accumulation and AD with amyloid-beta (Aβ) clearance failure [36, 86]. In breast and nasopharyngeal cancers, LAPTM4A promotes TAM polarization through NF-κB hyperactivation and lysosomal pH elevation, reflecting LAPTM4A-mediated neuroinflammatory cascades in amyotrophic lateral sclerosis (ALS) microglia [105]. Furthermore, LAPTM5’s role in disrupting endosomal trafficking, evident in pancreatic cancer invasion, parallels its involvement in IRI, where impaired autophagic flux exacerbates neuronal death.

The mTORC1/LAPTM4B axis in cancer elucidates how LAPTM4B overexpression sustains lysosomal mTORC1 signaling, inhibiting autophagosome maturation. This mechanism is directly translatable to the proteostasis collapse observed in AD and PD, where inhibiting the identical pathway restores lysosomal acidification. The stabilization of NF-κB by LAPTM4A in glioma-associated macrophages aligns with neuroinflammatory profiles. Moreover, cancer-derived inhibitors such as chloroquine analogs demonstrate promise in preclinical models of ALS [106]. Mutations in the dileucine motif of LAPTM5, which disrupt endosome-lysosome fusion, have been characterized in colorectal cancer metastasis, providing a structural rationale for neuronal vulnerability to IRI. These parallels validate the use of cancer models as high-throughput platforms for exploring LAPTM-related neural mechanisms, thereby circumventing ethical constraints associated with primary neuron studies.

Cancer therapeutic strategies that target LAPTM lysosomal functions offer immediately translatable interventions for neurological disorders, utilizing established pharmacological agents and delivery systems. Knocking out LAPTM4A/B via CRISPR or siRNA has been shown to restore autophagic flux and sensitize HCC and GBM to chemotherapy [107]. This approach also applies to neurodegeneration, as the depletion of LAPTM4A reduces tau hyperphosphorylation and α-synuclein aggregation in induced pluripotent stem cell (iPSC)-derived neurons [108]. Inhibition of LAPTM4B using chloroquine and bafilomycin A1 reverses V-ATPase displacement in breast cancer, restoring lysosomal acidification; similar repositioning in PD mouse models significantly reduces dopaminergic cell loss. Furthermore, overexpression of LAPTM5 through AAV-mediated delivery enhances autophagosome-lysosome fusion in pancreatic cancer resistance models, reflecting neuroprotective effects in IRI where LAPTM5 upregulation preserves cortical neuron survival post-stroke.

Combination strategies further expedite translation, as cancer mTORC1 inhibitors (rapamycin analogs) synergize with LAPTM4A siRNA to reprogram TAMs in GBM, paralleling the repolarization potential of microglia in neuroinflammation. Nanoparticle delivery systems validated in brain tumors ensure CNS penetration for these interventions. This evidence, initially derived from general cancer research, positions LAPTM proteins as mechanistic accelerators with therapeutic potential for neurological disorders—an assertion already well-established in the context of PD, AD, GBM, and various other nervous system conditions.

### Challenges and future research directions

Despite the promising therapeutic landscape surrounding LAPTM proteins, critical gaps remain. Current data predominantly focus on tumor and immune cells, overlooking neuron-, astrocyte-, and oligodendrocyte-specific functions. There is a need for single-cell transcriptomics and spatial multi-omics to develop comprehensive neural atlases. Integrated multi-disease models must differentiate shared mechanisms (such as energy metabolism and ferroptosis/pyroptosis) from disorder-specific pathways across gliomas, ischemia, and neurodegeneration.

Large-scale population studies assessing LAPTM polymorphisms in relation to disease risk and treatment efficacy, alongside multi-center clinical trials, are essential [91]. The challenge of crossing the blood-brain barrier remains paramount, necessitating the development of nanoparticle, exosome, and trans-blood–brain barrier (BBB) platforms for LAPTM regulators. Validating LAPTM agonists and inhibitors in humanized neural systems will be crucial for bridging the gap between preclinical promise and clinical application.

## Conclusion

This synthesis positions LAPTM proteins as master regulators of lysosomal homeostasis, with their dysregulation linking various neurological disorders, including gliomas, ischemia, neurodegeneration, and neuroinflammation. Their central role in autophagy, immunity, lipid metabolism, and proteostasis suggests that LAPTM modulation represents a convergent therapeutic paradigm. Gene therapy, immunotherapy synergies, and biomarker strategies hold immediate translational promise. Addressing cell-specificity issues, delivery challenges, and validation obstacles through multi-omics and precision trials will unlock LAPTM’s full potential, transforming lysosomal biology into clinical reality for patients facing these debilitating conditions.
